# Lower ratio of high-molecular-weight adiponectin level to total may be associated with coronary high-risk plaque

**DOI:** 10.1186/1756-0500-6-83

**Published:** 2013-03-06

**Authors:** Masao Moroi, Shamima Akter, Ryo Nakazato, Taeko Kunimasa, Hirofumi Masai, Tatsuhiko Furuhashi, Hiroshi Fukuda, Eiichi Koda, Kaoru Sugi, Subrina Jesmin

**Affiliations:** 1 Department of Cardiology, National Center for Global Health and Medicine, Toyama 1-21-1, Shinjuku-ku, Tokyo 162-8655, Japan; 2 Cardiac Imaging, Cedars-Sinai Medical Center, Los Angeles, CA, USA; 3 Division of Cardiovascular Medicine, Toho University Ohashi Medical Center, Tokyo, Japan; 4 Department of Radiology, Toho University Ohashi Medical Center, Tokyo, Japan

**Keywords:** Adiponectin, High-molecular-weight adiponectin, Coronary artery plaque, Coronary low-attenuation plaque

## Abstract

**Background:**

Although high-molecular-weight (HMW) adiponectin is believed to protect against atherosclerosis, the association between HMW adiponectin and the composition of coronary plaques is unknown. We evaluated whether the HMW to total adiponectin ratio was associated with the presence of coronary plaque and its composition using multi-slice computed tomography coronary angiography (MSCTCA).

**Methods:**

Serum total and HMW adiponectin levels were measured in 53 consecutive patients (age, 71) with >50% coronary artery stenosis detected by MSCTCA. A low-attenuation coronary plaque was defined as a plaque with a mean CT density <50 Hounsfield units. Multivariate logistic regression analyses were performed to evaluate the predictors of the presence of low-attenuation coronary plaques, which is thought to be high risk, on CT.

**Results:**

Decreased serum levels of total as well as HMW adiponectin were significantly associated with the presence of at least one calcified or non-calcified coronary artery plaque (total adiponectin level: odds ratio 0.76, 95% CI 0.58–0.99, P = 0.048; HMW adiponectin level: odds ratio 0.65, 95% CI 0.42–0.99, P = 0.047). A low ratio of HMW to total adiponectin was significantly associated with the presence of low-attenuation coronary plaques (4.55, 1.94–21.90, P = 0.049). However, neither the total adiponectin nor the HMW adiponectin level was associated with the presence of low-attenuation coronary plaques.

**Conclusion:**

Lower total or HMW adiponectin levels are associated with the presence of calcified and non-calcified coronary plaques, whereas a lower ratio of HMW to total adiponectin associated with the presence of low-attenuation coronary plaques (thought to be high risk). Measurement of total and HMW adiponectin levels and the HMW to total adiponectin ratio may be useful for risk stratification of coronary artery plaques.

## Background

Atherosclerotic cardiovascular disease is the leading cause of death worldwide [1]. Despite major advances in the treatment of coronary heart disease patients, a large number of victims of the disease who are apparently healthy die suddenly without prior symptoms. Currently available screening and diagnostic methods are insufficient to identify victims before the event occurs. Recently, lesion composition rather than size has been emphasized in determining the acute complications of atherosclerotic disease in humans. Many studies have suggested that non-obstructive lipid-rich plaques with a thin cap are prone to rupture and result in acute coronary artery occlusions [2,3] whereas obstructive, fibrous plaques with a thick cap result in clinically stable angina pectoris.

A large number of blood biomarkers, such as high-sensitivity C-reactive protein (hsCRP), tumor necrosis factor alpha, and oxidized low-density lipoprotein (ox-LDL), involved in inflammation, oxidation, and lipid metabolism have been demonstrated to play an important role in atherogenesis [4]. Several of those biomarkers have also been shown to be independent predictors of cardiovascular events [5]. Recently, adiponectin, a protein secreted by adipose tissue, has been reported as an important mediator against the development of coronary atherosclerosis [6-8]. Serum adiponectin levels are markedly decreased in patients with visceral obesity and insulin resistance [9,10]. Low adiponectin levels have also been linked with coronary artery disease (CAD) [11] and have been shown to be a risk factor for cardiovascular events [12]. Low adiponectin levels are also independently associated with the development of coronary artery calcification (CAC) [13].

Adiponectin in particular is abundant in the circulation as a low-molecular-weight trimer and a medium-molecular-weight hexamer, but it also occurs in a high-molecular-weight (HMW) form [14]. The HMW isoform binds most avidly to its receptors and stimulates AMP-activated protein kinase, which is one of the key molecules mediating the metabolic actions of adiponectin. Recently, several papers have reported that HMW adiponectin is the more active form and that it has stronger anti-atherogenic effects than other types of adiponectin [15-18]. However, whether HMW adiponectin levels, total adiponectin levels, or the HMW to total adiponectin ratio is more important for vascular protection and which factor is the better predictor of the severity of coronary atherosclerosis remains unclear [7,19-21].

With advances in imaging of coronary atherosclerosis, angiographic evaluation of coronary luminal stenosis has become a surrogate marker of the severity and extent of atherosclerosis. However, conventional coronary angiography has important limitations, one of which is its low predictive value for assessing atherosclerotic plaque burden and for predicting acute coronary syndrome events [22,23]. In addition, it does not enable identification of non-obstructive coronary plaques or determination of the composition of atherosclerotic plaques. Recent advances in coronary imaging have opened another potential window to better understand the links between biomarkers predictive of disease presence and complications and the anatomic diagnosis of coronary atherosclerosis. With advances in contrast-enhanced computed tomography (CT) angiography, evaluation of CAC as well as the detection and quantification of non-calcified plaque components is now possible. To date, only two studies have investigated the association between coronary atherosclerotic plaques detected by CT angiography and adiponectin levels [18,24]. However, their results are inconsistent. Therefore, the purpose of this study was to evaluate the associations between serum HMW adiponectin levels and the HMW to total adiponectin ratio and the composition of coronary artery plaques detected by multi-slice CT coronary angiography (MSCTCA) in patients with suspected coronary artery disease.

## Methods

### Study patients

**Figure 1:**
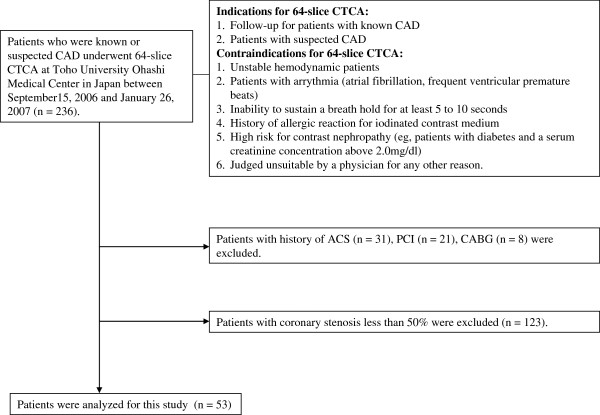
**Screening of study patients and indications for computed tomography coronary angiography.** CAD; coronary artery disease, CTCA; computed tomography coronary angiography, ACS; acute coronary syndrome, PCI; percutaneous coronary intervention, CABG; coronary artery bypass grafting.

Screening of study patients and indications for 64-slice CTCA were shown in Figure 1. Patients with known or suspected CAD were indicated for 64-slice CTCA. Patients with unstable hemodynamic situations, cardiac arrhythmias (i.e., chronic atrial fibrillation or frequent paroxysmal premature beats), inability to sustain a breath hold for at least 5 to 10 seconds, history of allergic reaction for iodinated contrast medium, high risk for contrast nephropathy (e.g., patients with diabetes and a serum creatinine concentration above 2.0 mg/dl) were contraindicated for 64-slice CTCA. A total of 236 consecutive patients with known or suspected CAD who underwent 64-slice CTCA at Toho University Ohashi Medical Center in Japan between September 15, 2006 and January 26, 2007 were screened for eligibility for this study. Written informed consent had been obtained from all patients. Patients with history of acute coronary syndrome (ACS, n = 31), percutaneous coronary intervention (n = 21), and/or coronary artery bypass grafting (n = 8) were excluded, because these patients were given medications such as statins, which may affect serum adiponectin levels. And 123 of the 176 remaining patients had normal coronary or coronary stenosis less than 50%. Consequently, a total of 53 patients with obstructive stable CAD were studied. The study was approved by the ethical committee of Toho University Ohashi Medical Center, Tokyo, Japan.

### Performance of 64-slice CTCA

Patients with a resting heart rate >60 beats/min received 1 mg/kg atenolol orally 60 min before imaging, and all received 0.3 mg nitroglycerin sublingually 5 min before imaging. A 64-slice CT machine (Aquilion 64; Toshiba Medical Systems, Otawara, Japan)was used with the following parameters: collimation 64 × 0.5 mm; detector pitch, 9.8–11.2; pixel size, 0.39 mm × 0.39 mm; gantry rotation time, 350 ms; tube current, 400 mA; and voltage, 120 kV. Contrast agent (320 mgI/ml^−1^; Optiray 320, Tyco Healthcare, Tokyo, Japan) was injected at a rate of 0.06 ml/kg/s during the entire scan time as well as for an additional 2 s, followed by administration of 0.15 ml/kg of contrast media plus 0.15 ml/kg of saline solution using a dual injector. Acquisition of the CT data and electrocardiography were started as soon as the signal density level in the ascending aorta reached a predefined threshold of 250 Hounsfield units (HU). The effective radiation dose was 15–18 mSv. Acquisition time was reduced to 200 ms by applying a half-scan algorithm (only data from a 180° gantry rotation was used for image reconstruction) in all patients. The reconstructed CT image data were transferred to a computer workstation for post-processing (ZIO M900; Amin/ZIO, Tokyo, Japan). Using this workstation, both cross-sectional and curved multiplanar reformation images were analyzed. These images were interpreted by an experienced cardiologist in conjunction with radiologists who were unaware of the patients’ detailed clinical backgrounds. We defined obstructive CAD on the CT images as stenosis >50% diameter.

### Assessment of coronary plaques

Plaques (calcified or non-calcified) were defined as structures >1 mm in diameter within the vessel wall adjacent to the coronary artery lumen but distinguishable from the perivascular tissues. A calcified coronary plaque was defined as a plaque with a CT density of >300 HU. We evaluated images with the optimal setting for detecting non-calcified coronary plaques and outer vessel boundaries. The setting was on average obtained at a width representing 155% of the mean intensity within the lumen and at a level representing 65% of the mean intensity, as reported previously [25]. A non-calcified coronary plaque was defined as a plaque with a CT density of ≤130 HU. A low-attenuation coronary plaque was defined as a plaque with a mean CT density of <50 HU within a non-calcified coronary plaque. A plaque with a mean CT density of <50 HU is considered a lipid-rich plaque (soft plaque) in IVUS imaging [26].

### Blood test

Blood samples were taken from all patients for lipid analysis when 64-slice CTCA was performed, and levels of fasting glucose, fasting insulin, low density lipoprotein-cholesterol (LDL-C), high density lipoprotein-cholesterol (HDL-C), triglyceride, HbA1c, and high-sensitivity C-reactive protein (hsCRP) were measured. Blood glucose and insulin levels were determined by glucose dehydrogenase method and chemiluminescent immunoassay, respectively. Serum levels of LDL-C, HDL-C, and triglyceride were determined by enzymatic colorimetric method. HbA1c levels were determined by latex agglutination-turbidimetric immunoassay, and serum hsCRP levels were determined by latex nepherometric method. The homeostasis model assessment index of insulin resistance (HOMA-IR) value was calculated as fasting glucose level (mg/dL) × fasting insulin level (μU/ml)/405. Total and HMW adiponectin were measured by an enzymed-linked immunosorbent assay (ELISA) method using a kit from Daiichi-kagakuyakuhin Co. (Tokyo, Japan). In brief, adiponectin circulates in plasma in 3 forms: trimer, hexamer, and in a HMW form: the HMW form consists of 12–18 subunits. Pretreatment with protease II was used to breakdown the trimers and hexamers, and the remaining HMW subunits were transformed to dimers by SDS buffer. Dimer levels were measured by ELISA.

### Statistical analysis

Descriptive characteristics for all variables are expressed as mean±SD for normally distributed continuous variables, medians with an interquartile range for non-normally distributed continuous variables, and percentages for categorical variables. Differences in characteristics between subjects with and without plaques (calcified and non-calcified) or between subjects with or without coronary low attenuation plaques were assessed using a chi-square test for categorical variables, a Mann–Whitney test, and a t-test as appropriate for non-normal and continuous variables. Univariate and multivariate logistic regression analyses were performed to assess associations between coronary plaques and total adiponectin levels, HMW adiponectin levels, and the HMW to total adiponectin ratio. Receiver-operating characteristic (ROC) analyses were performed to quantify the power of the total adiponectin level, HMW adiponectin level, and HMW to total adiponectin ratio for predicting the presence of coronary artery plaques. Optimal cut-off values for each of these factors were determined to provide the highest diagnostic accuracy for predicting the presence of calcified and non-calcified coronary plaques. In addition, to better understand the roles of adiponectin, HMW adiponectin, and the HMW to total adiponectin ratio in predicting the presence of low-attenuation coronary plaques, logistic regression was performed using the optimal cut-off values for all adipokines. Three models were considered for analysis. The first model was unadjusted. The second model was adjusted for age and sex. And the third model was additionally adjusted for HDL cholesterol. Two-sided P values of <0.05 were considered statistically significant. All statistical analyses were performed using STATA version 12.0 (Lakeway Drive, College Station, Texas USA).

## Results

### Coronary plaques and adiponectin levels

The clinical characteristics of the study subjects classified according to the presence and absence of plaques (calcified and non-calcified) or presence and absence of coronary low attenuation-plaques are shown in Table 1. No significant differences were observed between groups in sex, age, conventional coronary risk factors such as smoking, hypertension, and diabetes mellitus. Moreover, no significant differences were observed in serum LDL cholesterol, triglyceride, hemoglobin A1c levels, the HOMA-IR score, hsCRP, and body mass index (BMI). High-density lipoprotein (HDL) cholesterol levels were significantly lower in subjects with plaques compared with those without plaques (P = 0.02). No significant associations were observed for any of the covariates between coronary with and without low attenuation-plaque.

**Table 1:** Patients characteristic for the presence of plaque

	**Plaque (−)**	**Plaque (+)**	**P value**
	**Total plaque**		
Male/Female	6/4	31/12	0.45
Age (years)	71.4±8.7	70.7±8.6	0.80
Smoking (%)	50	42	0.64
Diabetes mellitus (%)	40	47	0.71
Hypertension (%)	78	89	0.34
Glucose (mg/dL)	110.0 (100.0-176.0)	116 (99.0-144.0)	0.50
LDL-Cholesterol (mg/dL)	88.5 (84.0-137.0)	108.0 (95.0-127.0)	0.35
HDL-Cholesterol (mg/dL)	59.0 (46.0-64.0)	47.0 (42.0-55.0)	0.02
Triglyceride (mg/dL)	114.5 (88.0-192.0)	132.0 (99.0-222.0)	0.50
HbA1c (mg/dL)	5.3 (4.9-6.5)	5.6 (5.1-6.5)	0.48
HOMA-IR	2.0 (1.5-8.8)	2.6 (1.6-7.9)	0.70
hsCRP (mg/dL)	0.03 (0.02-0.09)	0.06 (0.03-0.12)	0.12
BMI (kg/m^2^)	23.08±2.7	24.0±2.9	0.40
	**Low attenuation plaque**		
Male/Female	15/10	16/2	0.04
Age (years)	69.1±9.3	71.8±8.12	0.34
Smoking (%)	36	50	0.36
Diabetes mellitus (%)	44	50	0.70
Hypertension (%)	91	87	0.65
Glucose (mg/dL)	108.0 (100.0-139.0)	119.0 (94.0-148.0)	0.89
LDL-Cholesterol (mg/dL)	110.0 (99.0-132.0)	106.0 (85.0-127.0)	0.47
HDL-Cholesterol (mg/dL)	106.0 (85.0-127.0)	48.5 (42.0-56.0)	0.67
Triglyceride (mg/dL)	126.0 (94.0-222.0)	134.0 (115.0-195.0)	0.70
HbA1c (mg/dL)	5.5 (5.2-6.2)	6.2 (5.0-7.0)	0.82
HOMA-IR	2.4 (1.2-7.9)	4.0 (1.8-6.7)	0.30
hsCRP (mg/dL)	0.04 (0.03-0.10)	0.06 (0.03-0.12)	0.83
BMI (kg/m^2^)	23.06±2.5	24.06±2.8	0.46

LDL, low density lipoprotein; HDL, high density lipoprotein; HbA1c, hemoglobin A1c; HOMA-IR, homeostasis model assessment of insulin resistance; hsCRP, high-sensitivity C-reactive protein; BMI, body mass index.

**Figure 2:**
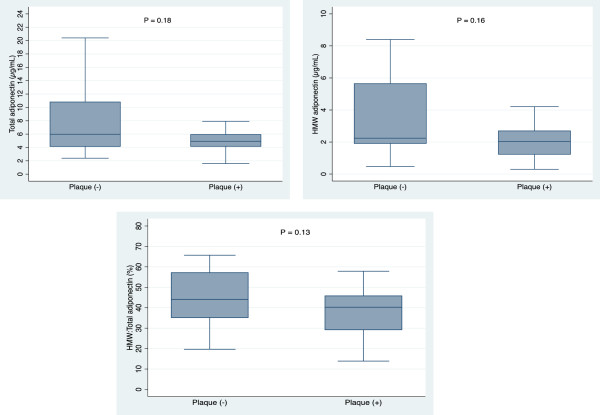
Box plots showing differences in the total adiponectin levels, high molecular weight (HMW) adiponectin levels, and HMW to total adiponectin ratio in patients with and without coronary artery plaques.

Figure 2 shows total adiponectin levels, HMW adiponectin levels, and the HMW to total adiponectin ratio in subjects with and without coronary plaques (calcified and non-calcified). No significant difference was observed between subjects with and without coronary plaques in terms of plasma total adiponectin levels, HMW adiponectin levels, or the HMW to total adiponectin ratio. However, there was a trend toward lower total adiponectin levels in subjects with coronary plaques.

**Figure 3:**
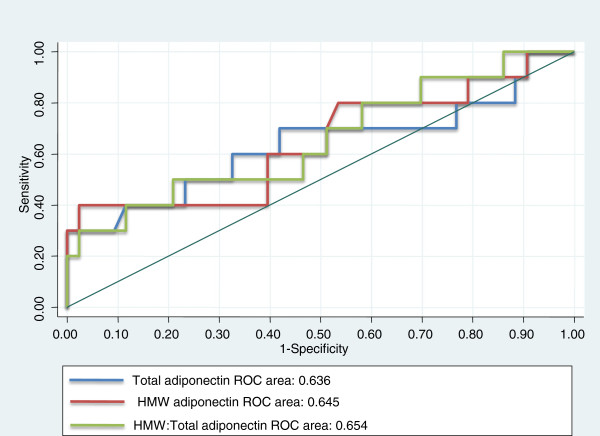
Receiver-operating characteristic curves of plasma levels of total adiponectin, high-molecular-weight (HMW) adiponectin, and the HMW to total adiponectin ratio and predictive values for the presence of coronary artery plaques.

Figure 3 shows the ROC curves for plasma total adiponectin levels, HMW adiponectin levels, and the HMW to total adiponectin ratio and predictive values for the presence of coronary artery plaques. The area under the curve (AUC) for total adiponectin levels (0.636), HMW adiponectin levels (0.645), and the HMW to total adiponectin ratio (0.653) was similar.

Table 2 shows the association of total adiponectin, HMW adiponectin, HMW to total adiponectin ratio with coronary plaques. In unadjusted model decreased total adiponectin and HMW adiponectin levels were significantly associated with the presence of coronary plaques. In age- and sex-adjusted model the association remained significant. Further adjustment for HDL cholesterol did not change the association. The fully adjusted odds ratios of total adiponectin levels (per 1.0 μg/ml) and HMW adiponectin levels (per 1.0 μg/ml) for the presence of coronary plaques were 0.76 (0.58–0.99) and 0.65 (0.42–0.99), respectively.

**Table 2:** Predictors for the presence of plaque

**Predictors**	**Odds ratio**	**95% CI**	**P value**^**a**^
Total adiponectin (μg/mL)			
Unadjusted	0.76	0.59-0.98	0.036
Age- and sex-adjusted	0.75	0.57-0.99	0.041
Multivariable-adjusted^b^	0.76	0.58-0.99	0.048
HMW adiponectin (μg/mL)			
Unadjusted	0.64	0.43-0.97	0.036
Age- and sex-adjusted	0.63	0.41-0.97	0.036
Multivariable-adjusted^b^	0.65	0.42-0.99	0.047
HMW/Total adiponectin			
Unadjusted	0.95	0.89-1.01	0.11
Age- and sex-adjusted	0.95	0.89-1.01	0.14
Multivariable-adjusted^b^	0.95	0.89-1.02	0.16

HMW adiponectin, high molecular weight adiponectin; CI, confidence interval.

^a^Based on logistic regression analysis.

^b^Additionally adjusted for HDL cholesterol.

### Predictors of low-attenuation coronary plaque

**Figure 4:**
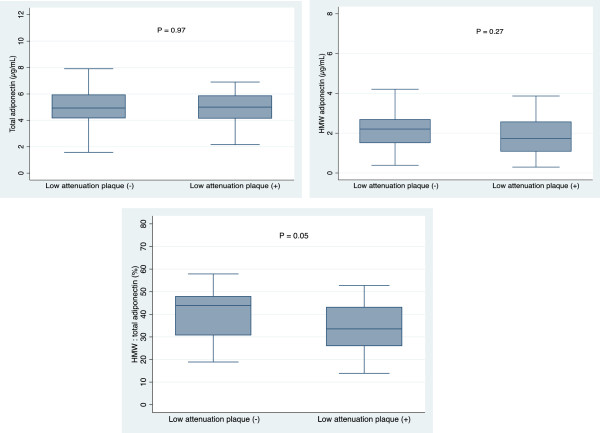
Box plots showing differences in total adiponectin levels, high-molecular-weight (HMW) adiponectin levels, and the HMW to total adiponectin ratio in patients with and without low-attenuation coronary plaques.

In our second series of analyses, we compared serum total adiponectin levels, HMW adiponectin levels, and the HMW to total adiponectin ratio between subjects with and without low-attenuation coronary plaques. Figure 4 shows total adiponectin levels, HMW adiponectin levels, and the HMW to total adiponectin ratio in subjects with and without low-attenuation coronary plaques. No significant differences were observed in plasma total and HMW adiponectin levels between subjects with and without low-attenuation coronary plaques. However, a significant difference was observed in the HMW to total adiponectin ratio between subjects with and without low-attenuation coronary plaques (median 44% and 33%, respectively, P = 0.05).

**Figure 5:**
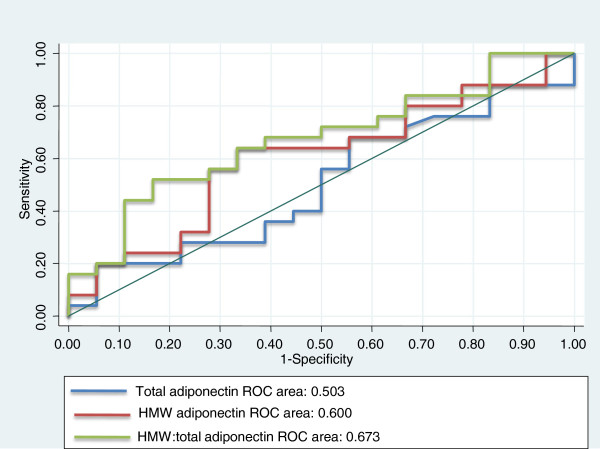
Receiver-operating characteristic curves of plasma levels of total adiponectin, high-molecular-weight (HMW) adiponectin, and the HMW to total adiponectin ratio and predictive values for the presence of low-attenuation coronary plaques.

Figure 5 shows the ROC curves of plasma total adiponectin levels, HMW adiponectin levels, and the HMW to total adiponectin ratio for predicting the presence of low-attenuation coronary plaques. AUC for the HMW to total adiponectin ratio was larger (0.636) than that for total and HMW adiponectin levels (0.50 and 0.60, respectively). The optimal cut-off values for total adiponectin levels, HMW adiponectin levels, and the HMW to total adiponectin ratio for the presence of low-attenuation coronary plaques were 4.3 μg/ml, 1.8 μg/ml, and 43.8%, respectively.

Table 3 shows the association of total adiponectin, HMW adiponectin, and HMW to total adiponectin ratio with coronary low-attenuation plaque. Multivariate logistic regression showed that the HMW to total adiponectin ratio, but not total or HMW adiponectin levels, was significantly associated with the presence of low-attenuation coronary plaques. In age- and sex-adjusted model subjects with a HMW to total adiponectin ratio of <43.8% had a higher risk (3.87 times) of having a low-attenuation coronary plaque than those with a higher ratio (P = 0.046). Further adjustment for HDL cholesterol did not change the association. Plasma total adiponectin levels of <4.3 μg/ml and HMW adiponectin levels of <1.8 μg/ml were not associated with the presence of low-attenuation coronary plaques.

**Table 3:** Predictors for the presence of low attenuation plaque

**Predictors**	**Odds ratio**	**95% CI**	**P value**^**a**^
Total adiponectin <4.3 μg/mL			
Unadjusted	1.06	0.29-3.86	0.93
Age and sex adjusted	1.03	0.26-4.12	0.96
Multivariable-adjusted^b^	0.94	0.23-3.82	0.94
HMW adiponectin <1.8 μg/mL			
Unadjusted	2.79	0.80-9.8	0.107
Age and sex adjusted	2.42	0.65-9.01	0.19
Multivariable-adjusted^b^	2.94	0.75-11.52	0.12
HMW/Total adiponectin <43.8%			
Unadjusted	5.42	1.25-23.49	0.024
Age and sex adjusted	3.87	1.82-18.14	0.046
Multivariable-adjusted^b^	4.55	1.94-21.90	0.049

HMW adiponectin, high molecular weight adiponectin; CI, confidence interval.

^a^ Based on logistic regression analysis.

^b^ Additionally adjusted for HDL cholesterol.

## Discussion

Our study was designed to investigate associations between levels of various forms of circulatory adiponectin and the presence of calcified and non-calcified coronary plaques in subjects with obstructive stable CAD. We also extended our analysis to include the presence of low-attenuation coronary plaques. We found that (1) circulatory total adiponectin and HMW adiponectin levels were significantly and inversely associated with the presence of calcified and non-calcified coronary plaques and (2) the HMW to total adiponectin ratio was significantly and inversely associated with the presence of low-attenuation coronary plaques in subjects with obstructive stable CAD. No significant association was observed between the HMW to total adiponectin ratio and the presence of calcified and non-calcified coronary plaques. Circulatory levels of HMW and total adiponectin are important predictors of the presence of calcified and non-calcified coronary plaques, whereas the HMW to total adiponectin ratio is important for predicting the presence of low-attenuation coronary plaques. Furthermore, in the present study subjects with obstructive stable CAD, none of the traditional demographic or clinical parameters, except for plasma HDL levels, was useful for predicting the presence of calcified and non-calcified coronary plaques or low-attenuation coronary plaques.

In our study, lower plasma HDL levels were significantly associated with the presence of calcified and non-calcified coronary plaques. In a prospective observational cohort study, only diabetes and the HDL level, but not other factors such as hypertension, hypercholesterolemia, hypertriglyceridemia, and hyper-LDL cholesterolemia, were associated with the presence of angiographically obstructive CAD [27]. Another study investigating a significant association between the HDL level and CAC demonstrated that BMI, fasting glucose levels, fasting triglyceride levels, HDL cholesterol levels, and blood pressure clustered together under a single latent factor, and that this latent factor was associated with CAC [28]. Therefore, even if other clinical parameters were not significantly associated with the presence of coronary artery plaques, the HDL level could be an important background characteristic for the development of coronary plaques in subjects with obstructive stable CAD.

In a previous study, low circulatory levels of adiponectin were found to be an independent predictor of the presence of calcified and non-calcified coronary atherosclerotic plaques [24]. Another study [29] showed that higher plasma adiponectin levels were associated with a lower risk of ACS, suggesting that the pathophysiological role of adiponectin may be related to the stability of atherosclerotic plaque. Consistent with these studies, we found by multivariable logistic regression that a higher total adiponectin level was associated with a lower risk of the presence of coronary atherosclerotic plaque. On the other hand, another study showed no significant association between total adiponectin levels and coronary artery plaques [18]. Differences in the characteristics of the study subjects and in sample size might explain this discrepancy. In addition, we found a similar association between HMW adiponectin levels and the presence of coronary plaques in our study subjects. In a recent study, HMW adiponectin has been suggested to be a better predictor of coronary artery plaque characteristics and the progression of atherosclerosis than total adiponectin levels [18]. Previous studies have indicated that HMW adiponectin is associated with the extent of CAD [30] and increased arterial stiffness [16] and concluded that HMW adiponectin might have higher biological activity than other types of adiponectin. In our study HMW and total adiponectin might have a similar power for predicting the presence of coronary artery plaques in subjects with obstructive stable CAD.

In our study, although total and HMW adiponectin levels were not associated with the presence of low-attenuation coronary plaques, a lower ratio of HMW to total adiponectin was associated with the presence of coronary low-attenuation plaques. In a previous study, both HMW adiponectin levels and the HMW to total adiponectin ratio were found to be inversely correlated with the extent of CAD [30]. Another study showed that a lower ratio of HMW to total adiponectin was associated with CAD in patients with diabetes [19]. However, von Eynatten et al. reported that neither the total adiponectin nor the HMW adiponectin level was an independent predictor for cardiovascular events in patients with CAD [21]. Thus, there is currently no consistent evidence for determining whether total adiponectin levels, HMW adiponectin levels, or the HMW to total adiponectin ratio is a more reliable marker for predicting CAD severity. The inverse association of HMW to total adiponectin ratio with the presence of coronary low-attenuation plaques supports the findings of Liang et al. who demonstrated that a lower ratio of HMW to total adiponectin was associated with the severity of angiographic coronary atherosclerosis [31]. Our ROC analysis also demonstrated that the predictive power of the HMW to total adiponectin ratio tended to be higher than that of the total and HMW adiponectin level for predicting the presence of low-attenuation coronary plaques.

Our study has several limitations. First, the sample size was relatively small. Second, the effects of potential confounding factors such as hsCRP, insulin resistance, and other adipokines have not been completely evaluated in terms of the association between adiponectin and coronary artery plaques. Third, diabetic subjects receiving Pioglitazone may bias our results due to possible effects of medication on adiponectin levels and coronary artery plaques. However, the number of patients taking Pioglitazone was very few in our study subjects (4 subjects are taking Pioglitazone out of 24 diabetic patients), so their impact on results was very low. Finally, our study subjects were not a random sample of invasive coronary angiography-based CAD patients, but a consecutive sample of patients with CT-based obstructive stable CAD. Caution is required when generalizing the present results to suspected cases of CAD as well as to patients with ACS.

## Conclusion

In conclusion, in our patients with obstructive stable CAD, lower plasma levels of total and HMW adiponectin were associated with the presence of calcified and non-calcified coronary artery plaques. On the other hand, a lower ratio of HMW to total adiponectin was associated with the presence of low-attenuation coronary plaques. Therefore, total and HMW adiponectin levels are important markers for predicting the presence of coronary artery plaques, and the HMW to total adiponectin ratio is important for predicting the presence of low-attenuation coronary plaques. Further large-scale, prospective clinical studies are warranted to elucidate the underlying mechanism.

## Abbreviations

ACS: Acute coronary syndrome; AUC: Area under the curve; BMI: Body mass index; CAC: Coronary artery calcification; CAD: Coronary artery disease; CI: Confidence interval; CT: Computed tomography; HDL: High-density lipoprotein; HMW: High-molecular-weight; HOMA-IR: Homeostasis model assessment index of insulin resistance; hsCRP: High-sensitivity C-reactive protein; HU: Hounsfield units; LDL: Low density lipoprotein; MSCTCA: Multi-slice computed tomography coronary angiography; ROC: Receiver-operating characteristic

## Competing interests

The authors declare that they have no conflicts of interest.

## Authors’ contributions

MM contributed to conception, research design, and data acquisition; NR, KT, MH, FT, and FH contributed to performance of CT coronary angiography and data acquisition; KE managed CT coronary angiography; SK managed biomarker measurement; AS helped in data analysis and interpretation; AS and MM drafted the manuscript; JS gives critical comments on revising the manuscript. All authors involved in substantially revising the manuscript and approve the final version of the manuscript. None of the authors had a conflict of interest.
